# Characterization and Long-Term Stability of Historical PMMA: Impact of Additives and Acrylic Sheet Industrial Production Processes

**DOI:** 10.3390/polym12102198

**Published:** 2020-09-25

**Authors:** Sara Babo, Joana Lia Ferreira, Ana Maria Ramos, Anna Micheluz, Marisa Pamplona, Maria Helena Casimiro, Luís M. Ferreira, Maria João Melo

**Affiliations:** 1 Department of Conservation and Restoration and Research Unit LAQV-REQUIMTE, NOVA School of Sciences and Technology (FCT NOVA), 2829-516 Caparica, Portugal; mjm@fct.unl.pt; 2 Department of Chemistry and Research Unit LAQV-REQUIMTE, NOVA School of Sciences and Technology (FCT NOVA), 2829-516 Caparica, Portugal; ana.ramos@fct.unl.pt; 3 Conservation Science Department, Deutsches Museum, Museumsinsel 1, 80538 Munich, Germany; a.micheluz@deutsches-museum.de (A.M.); m.pamplona@deutsches-museum.de (M.P.); 4 Center for Nuclear Sciences and Technologies (C2TN), Instituto Superior Técnico (IST), Universidade de Lisboa, 2695-066 Bobadela LRS, Portugal; casimiro@ctn.tecnico.ulisboa.pt (M.H.C.); ferreira@ctn.tecnico.ulisboa.pt (L.M.F.); 5 Department of Nuclear Sciences and Engineering (DECN), Instituto Superior Técnico (IST), Universidade de Lisboa, 2695-066 Bobadela LRS, Portugal

**Keywords:** poly(methyl methacrylate), PMMA, photodegradation, accelerated aging, production processes, additives, cadmium red, conservation

## Abstract

This work aims at understanding the influence of the production processes and materials in the properties and long term behavior of acrylic sheet, i.e., poly(methyl methacrylate) (PMMA), a material generally considered very stable in museum collections. A comparative study was conducted in samples from cast acrylic sheets produced in the early 2000s, from which manufacturing details were known, and samples provided by the artist Lourdes Castro from acrylic sheets she had bought in the 1960s. Transparent and red opaque cast acrylic samples, containing cadmium red pigment, were used. All samples were artificially aged in a solarbox with irradiation λ > 300 nm for a total period of 8000 h, and alterations were followed by a multi-analytical approach which included Raman, infrared (FTIR-ATR) and UV-Vis spectroscopies; gravimetry; size exclusion chromatography (SEC); thermogravimetry (TGA); micro-indentation; colorimetry; and optical microscopy. Not all cast PMMA sheets presented similar stabilities. We have concluded that the production processes (which may include the polymerization conditions, the organic additives and the origin of the monomer) play a more important role in the properties and long-term behavior of these acrylic sheets than the presence of cadmium red and/or the age of the material.

## 1. Introduction

Acrylic sheet, which consists of almost pure poly(methyl methacrylate) (PMMA), was developed industrially in the 1930s as the first “organic glass” [1,2]. Its remarkable optical properties, stiffness, light weight, mechanical and weathering resistance, ability to be easily thermoformed, and its capacity to be produced in different colors and transparency/opacity levels, made this material suitable for countless applications, such as airplane cockpits, architectural roofing systems, illuminated signs, sanitary furniture, or sophisticated design objects, just to name a few examples [3,4].

The properties of PMMA sheets were also attractive to artists, who began to incorporate this material in their artistic production, acquiring it from international recognized industrial companies or from micro-size enterprises, especially from the 1960s onwards when plastics in general became more available and widely spread in society [5,6]. In the conservation field, PMMA is considered a very stable plastic in opposition to other more instable plastics such as cellulose acetate, cellulose nitrate, plasticized poly(vinyl chloride) (PVC), and polyurethane [7,8,9,10]. However, there is still much to be known on how intrinsic factors, related with the materials and methods used by different producers, may impact on PMMA stability and lifetime. Following our previous research [11] our aim was to understand which intrinsic factors—e.g., the temperature and completeness of the polymerization process; the origin of the monomer; and the presence of additives, including an inorganic colorant (cadmium red)—may have a significant influence on the properties and susceptibility of PMMA sheets to photooxidation.

The main aging mechanism of PMMA, photooxidation, has been extensively studied [12,13,14,15,16,17,18,19,20,21,22,23]. The photodegradation mechanism of methacrylic and acrylic polymers has been followed by size exclusion chromatography and a distinction in behavior has been observed [22,24,25]. In both, scission and crosslinking are present, with scission being the dominant mechanism in acrylics, whereas in methacrylics crosslinking may compete depending on the volume of the side group, and it is therefore residual in PMMA (the methacrylic with the smallest side group, a methyl). Films and sheets of PMMA should absorb bellow 260 nm [13,15,16,17,18,19,20]; however, chromophores such as hydroperoxide groups are usually present in the polymer matrix as consequence of synthesis and processing, which may absorb radiation at wavelengths higher than the polymer and initiate photodegradation reactions [26]. The singularity of the stability of PMMA may be further explained by the fact that, to obtain the excellent optical properties typical of an acrylic sheet, the level of impurities must be extremely low [27]. Moreover, these impurities, or other chromophore species responsible for the initiation of photooxidation processes, do not have a catalytic effect. According to Sirampiguer and co-workers [19], alcoholic groups are formed as PMMA photooxidation products. These authors also suggested that in PMMA cast sheets of 3–6 mm thickness, the photooxidation would take place only in a superficial layer of ca 500 μm, because of the low permeability of oxygen in PMMA [19].

Most of the photodegradation studies on PMMA have been performed on pure polymer films prepared in laboratory, sometimes with controlled addition of other compounds/additives, and typically with radiation at λ < 300 nm to accelerate degradation. Fewer studies [19,28,29,30] have been conducted with commercial PMMA sheets and with radiations more similar to what an artwork may be exposed in real life.

It is also known that pigments can play a major role in the stability of a polymer matrix. Regarding photostability, pigments can either induce a protective effect by absorbing and/or screening UV light, or catalyze/accelerate the photochemical breakdown of the polymer by being photoactive [9,31]. Cadmium reds consist of cadmium sulfide co-precipitated with selenium, (*CdS_1-x_*Se*_x_*) [32,33]. In the coloring of plastics, cadmium pigments are commonly used together with BaSO_4_ in the form of lithopones, allowing the reduction of costs while maintaining good color values and stability [32,34]. In a recent work, the identification of this family of pigments in historical PMMA samples has been investigated [35], but to the best of our knowledge, studies that correlate their presence with the photostability of PMMA are still missing.

### Industrial Production Processes of Cast Sheets

PMMA sheets may be cast or extruded. Extruded sheets only became available in the late 1970s [3,36] and are of poorer quality than cast sheets [36]. The process of producing cast sheets is well described in several references [4,37,38] and consists basically in introducing monomer (or a pre-polymerized syrup of monomer), initiator, and other additives into glass molds. The filled molds are heated at controlled temperatures for polymerization of the methyl methacrylate (MMA). The duration of the process may vary from few hours to days depending on the sheet thickness and the technology used.

In our previous work [11], detailed information was collected about the production process of acrylic sheet in two different companies operating in Portugal between 1960s–2000s, Plásticos do Sado and Paraglas. These two companies produced exclusively cast PMMA sheets through radical initiated bulk polymerization. Even so, different methods were followed at each plant, which may have influenced the properties of the final material. The main aspects are summarized in Table 1.

Comparing the processes used by the two companies, the one used by Paraglas most probably presented advantages concerning the final quality of the material produced, namely:

1. Use of pure monomer, assuring less contaminants in the final product. Even though feedstock recycling of PMMA through pyrolysis is a well-established method [42,43], the purity of the material obtained may be affected by the presence of water and the composition of the scrap used [44]. Therefore, acrylic produced from it may present inferior properties compared to that prepared from neat MMA [45,46].
2. Better control of the uniformity of the sheets thickness, as the glass molds were hold against rigid metal surfaces.
3. Post-polymerization at 120 °C. In the first step of polymerization, conversion only reaches 80% to 90% because glasslike solidification of the reaction mixture occurs. To assure full transformation of the monomer into PMMA, it is necessary to raise the temperature above its glass transition temperature (*T*_g_) [38].

Differences in the molecular and physical properties between PMMA sheets produced by these two companies have been revealed for the first time by our preliminary studies [11]. In the present work, this research is continued by further exploring the characterization of the samples, which includes identification of the additives, and by conducting an accelerated aging experiment in order to investigate the impact of the differences found on their long term stability. Samples from transparent and red acrylic sheets produced by the two Portuguese companies (no longer operating but from which manufacturing details are known) and samples from PMMA sheets provided by the artist Lourdes Castro (Plexiglas^®^ and Altuglas^®^) were used. Lourdes Castro (b. 1930) is a Portuguese artist, who explored the possibilities given by acrylic sheet in her artworks between 1964 and 1968, while working in Paris [5,47]. The artist refers to the material she used as “plexiglas” (one of the most famous names by which cast PMMA sheet was commercialized) even though she has used both Plexiglas^®^ and Altuglas^®^, which she believed were the best quality acrylic sheets she could buy [12]. Plexiglas^®^ was the name of the material produced by the German company Röhm und Haas, but it was also used for the material produced in France by Alshtom/Altulor until 1962, through an agreement with the German company. It was only in 1962 that PMMA sheets produced by Altulor started to be named Altuglas^®^ and to compete in the market with the German Plexiglas^®^ [48], which may explain the preponderance of the name. Even though production details about Lourdes Castro’s PMMA samples were unknown and these samples had already more than 50 years of natural aging, they were closer to real cases in Museums and we expected to gain more knowledge about them by comparison with the Portuguese ones. Therefore, all samples were subjected to irradiation λ > 300 nm during 8000 h. Long periods of artificial aging are especially important in the conservation field, since artworks are expected to last for centuries. Alterations on the samples were followed by a multi-analyical approach, which included gravimetry; Raman, infrared (FTIR-ATR) and ultraviolet and visible (UV-Vis) spectroscopies; size exclusion chromatography (SEC); thermogravimetry (TGA); micro-indentation; colorimetry; and optical microscopy. Results are presented and discussed, attempting to correlate the different behaviors observed with the differences in their compositions and industrial production processes used. As it will be shown in this work, not all the cast acrylic sheets are alike and present the same stability.

## 2. Materials and Methods

### 2.1. Samples

Samples from six different cast acrylic sheets were used in this study (Table 2). Pieces of transparent and red acrylic were kindly provided by Lourdes Castro and, according to the artist, both are dated from the 1960s and identified as Plexiglas and Altuglas, respectively. Transparent sheets and red samples from color swatches, dated from 2000s and produced by Plásticos do Sado and Paraglas, were selected from the material archive of the Conservation and Restoration Department FCTNOVA and used also in this study for comparison. Selection of the two red samples from the color swatches was based on similarity of chemical composition with the one provided by Lourdes Castro [11]. The Plásticos do Sado and Paraglas samples were fundamental for this research because production details were known based on our previous work as described in the Introduction above.

Samples of 15 × 15 mm^2^ and 5 × 5 mm^2^ were cut from the different PMMA sheets by a 3D carving machine (Carvey^®^ by Inventables, Chicago, IL, USA) with a 300 W DC spindle of 12,000 rpm and 1/16” solid carbide bit, using the following cut settings: 1219.2 mm/min feed rate, 228.6 mm/min, and 0.5 mm of depth per pass. All samples were cleaned from dust and grease from handling in an agitated bath at room temperature of a mixture of 1% neutral detergent (Neutracon^®^) in distilled water; followed by rinsing with at least 3 agitated baths of distilled water. Excess water was removed with absorbent paper and samples were left to dry in a desiccator with silica gel.

### 2.2. Artificial Aging and Characterization Procedure

To compare the behavior of the different PMMAs under study, samples were artificially aged in a Solarbox 3000e accelerated aging apparatus (CO.FO.ME.GRA) equipped with a Xenon-arc light source filtered to λ > 300 nm, with constant irradiation of 800 W/m^2^ and black standard temperature varying between 60 to 100 °C (air temperature measured inside the chamber <40 °C), for a maximum period of 8000 h (total irradiance = 22,943 MJ/m^2^).The UV-Vis transmission spectrum of the filter used in the solarbox is presented in Supplementary Information. Samples were analyzed before irradiation and after 250, 500, 1000, 2000, 4000, 6000 and 8000 h. The 15 mm side samples (in triplicates of each PMMA typology, except RPS and RPA) were used for the non-destructive analysis methods (gravimetry, optical microscopy, colorimetry, infrared, Raman and UV-Vis spectroscopies) and returned to the solarbox after analysis. The series of 5 mm samples were used for the destructive methods (micro-indentation, size exclusion chromatography and thermogravimetry), therefore, at each time, two samples of each PMMA type were removed from the solarbox for analyses.

### 2.3. Analytical Methods

#### 2.3.1. Optical Microscopy (OM)

Optical microscopy (OM) was used to characterize visually the surfaces of the samples and detect eventual alterations. Images were acquired using a Zeiss Axioplan 2 Imaging system (Oberkochen, Germany), equipped with halogen (HAL 100) and mercury (N HBO 103) illuminators, coupled to Nikon DXM1200F microscope camera and ACT-1 control software (Tokyo, Japan). Microphotographs were acquired at all aging intervals using reflected light in bright field mode, at 50× and 500× magnifications. When necessary to examine some alterations, different magnifications and illumination modes were also used, including transmitted light, dark field and cross polarizing filters. Fluorescence microscopy images were acquired with blue-violet light, using the Zeiss Filter set 05 (excitation BP 395–440 nm, beamsplitter FT 460 nm, emission LP 470 nm).

#### 2.3.2. Color Measurements

Color determinations were made using a Datacolor International colorimeter (Microflash). The optical system of the measuring head uses diffuse illumination from a pulsed Xenon-arc lamp over an 8 mm-diameter measuring area. The color measurements were performed using the standard illuminant D65 and the CIE 1964 standard colorimetric observer (10°) geometry. Before color determinations, calibration was performed with bright white and black standard plates. Both the equipment measuring head and the sample were positioned in a costume made positioning mask, which allowed future measurements on exactly the same area. A white base composed of several layers of lab filter paper was used for all measurements and stored in the dark while not in use. For each determination of L*, a* and b* color coordinates, the mean value and standard deviation of three independent measurements over the same measurement area on the three sample replicates were calculated (nine readings), except for RPS and RPA which do not have replicates (three readings). Color variation in samples during artificial aging was calculated according to the CIE 1976 (CIELAB) color difference (∆E*) expression [49]:

∆E* = [(∆L*)^2^ + (∆a*)^2^ + (∆b*)^2^]^1/2^, (1)

in which ∆L* is the variation in Lightness, ∆a* the variation in the red/green color coordinate, and ∆b* the variation in the blue/yellow color coordinate.

#### 2.3.3. Gravimetry

Weight measurements were performed in a Sartorious CP225 D micro analytical balance (Göttingen, Germany). At each irradiation time, the mean value of three independent weight measurements was calculated and mass loss determined by subtracting the corresponding value obtained at *t* = 0 h. Final mass loss corresponds to the mean of the mass loss values of the three replicates for each typology (includes nine readings) converted to percentage of total initial weight. Samples were kept in a desiccator after irradiation until weight measurements were performed.

#### 2.3.4. UV-Vis Spectroscopy

Ultra-violet and Visible spectroscopy was carried out both in transmittance and reflectance modes, given that both transparent and opaque samples were under study. Spectra obtained in transmittance mode were collected using an Agilent Cary 5000 UV-Vis-NIR spectrophotometer (Agilent Technologies, Santa Clara, CA, USA), version 1.12, in a spectral range from 250–800 nm, at a scan rate of 600 nm/min, data interval of 1 nm and acquisition average time of 0.1 s. PMMA samples were placed in a specially designed holder (to ensure analysis was always performed in the same area) and analyzed in 0° angle (perpendicular to the light beam). Spectra obtained in reflectance mode were collected in a Shimadzu UV2501PC spectrophotometer (Kyoto, Japan) equipped with an integrating sphere, in a spectral range from 240–900 nm, at very slow option scan rate, and data interval of 1 nm. BaSO_4_ was used as reference sample. All spectra were collected as absorption (Abs) spectra.

#### 2.3.5. Infrared Spectroscopy in Attenuated Total Reflectance Mode (ATR-FTIR)

ATR-FTIR spectroscopy was carried out using an Agilent Handheld 4300 FTIR spectrophotometer, equipped with a ZnSe beam splitter, a Michelson interferometer and a thermoelectrically cooled DTGS detector. All spectra were acquired with a diamond ATR module, 64 scans and 4 cm^−1^ resolution. Three independent spectra were collected for each sample. All spectra are presented as acquired, without baseline corrections or other treatments except normalization to the carbonyl peak intensity, allowing a direct comparison of relative intensities to be made.

#### 2.3.6. Raman Spectroscopy (µ-Raman)

Raman spectroscopy was carried out using a Labram 300 Horiba JobinYvon spectrometer (Kyoto, Japan), equipped with a He–Ne 17 mW laser operating at 632.8 nm. The system was calibrated using a silicon standard. The laser beam was focused with a 50× Olympus objective lens. The laser power at the surface of the samples was controlled with neutral density filters. Raman data analysis was performed using LabSpec 5 software. All spectra are presented as acquired without any baseline correction or other treatment except normalization.

#### 2.3.7. Size Exclusion Chromatography (SEC)

Molecular weight distributions were determined with a Knauer Smartline system composed by a model 3800 autosampler (Berlin, Germany), a 1000 pump, and a 2300 refractive index detector. Data was collected with DataApex Clarity software, version 5.0.5.98. Separation was performed by two Waters Styragel HR 5E columns (Prague, Czech Republic), with 7.8 mm × 300 mm, and 5 µm particle size, after a Waters Styragel pre-column. Sample solutions in tetrahydrofuran (THF anhydrous >99.9%, Sigma-Aldrich, St. Louis, MO, USA) at approximately 0.15% (*w*/*v*) concentration were prepared at room temperature and filtered with 0.45 μm pore filters. Distilled THF stabilized with butyl-hidroxytoluene (BHT) was used as eluent at a flow rate of 1ml/min; the operating temperature was 30 °C. Column calibration was performed with monodispersed PMMA standards from Polymer Laboratories (*M*_p_: 1.14 × 10^3^ to 1.25 × 10^6^). Molecular weight distribution values, including weight-average molecular weight (*M*_w_), number-average molecular weight (*M*_n_), and molecular-weight dispersity (*Đ*_M_), were determined with the software DataApex Clarity, version 7.4, using the GPC analysis extension. Material for analysis was collected with a scalpel from the surface of the PMMA samples. At least two analyses, with independent sample collection and preparation, were performed to verify the consistency of the results.

#### 2.3.8. Micro-Indentation

Vickers micro-hardness measurements were performed on an Indentec (Zwick/Roell, Ulm, Germany) ZHμ Micro Hardness Tester. Vickers hardness (HV) is the quotient obtained by dividing the load applied by the square area of indentation, according to:

(2) $$\mathrm{HV}=1.854\times \frac{F}{d^{2}},$$

where *F* is the load in kgf and *d* the arithmetic mean of the two diagonals in mm. In this study, the load used was 0.3 kgf and the application time was 15 s. For each sample, the values presented result from the arithmetic mean of five different HV measurements, performed at a distance >5*d* from each other. Due to the influence of relative humidity in the HV, samples collected at each aging time were kept in a desiccator and measured in a row in consecutive days at the end of the experiment.

#### 2.3.9. Thermal Analysis

Thermal analysis was performed by thermogravimetry (TGA). Measurements were carried out in a TGA Q500 (TA Instruments, New Castle, DE, USA), in nitrogen atmosphere, from 25 to 500 °C, at a rate of 10 °C/min. All PMMA typologies under study were analyzed before accelerated aging; analysis during aging was only conducted in the transparent samples (TPS, TPA, and TPL) after 0, 500, 2000, 8000 h of irradiation. At least three TGA measurements were performed for each sample tested.

#### 2.3.10. Thermodesorption-Gas Chromatography/Mass Spectrometry (TD-GC/MS)

Additive characterization was performed using a Multi shot Pyrolyzer EGA/PY-3030D (Frontier Lab.) coupled with a 7890 B gas chromatograph and a 5977 B MSD mass spectrometer (both Agilent Technologies, Santa Clara, CA, USA). Around 200 µg of sample was added directly into a stainless steel Eco-cup sample holder (Frontier Lab). The thermodesorption was carried out from 50 (hold for 30 s) to 250 °C (hold for 3 min) with an increasing ratio of 20 °C/min and cryo-trap for the volatile focalization. GC separations were performed using a Frontier UA5 capillary column (30 m–0.25 F, 30 m × 250 µm × 0.25 µm, Frontier Lab.), Helium as a carrier gas at the flow rate of 1.2 mL/min, and a split ratio of 30:1. The injector temperature was set to 300 °C. The column temperature program was set from 40 °C (hold for 2 min), increasing rate of 20 °C/min until 280 °C (hold for 15 min). The ionization mode was electron impact at 70 eV in positive mode, the transfer line at 280 °C, the ion source temperature at 230 °C and a scanning mass range of 29 to 550 m/z. MSD ChemStation (Agilent Technologies) software was used for data analysis and the compounds were identified by interpretation of their EI mass spectra and comparison to NIST MS Search 2.2 and F-Search 3.5.0 (Frontier Lab) databases.

## 3. Results and Discussion

### 3.1. Characterization of the Test Samples

#### 3.1.1. Optical Microscopy and Colorimetry

**Figure 1:**
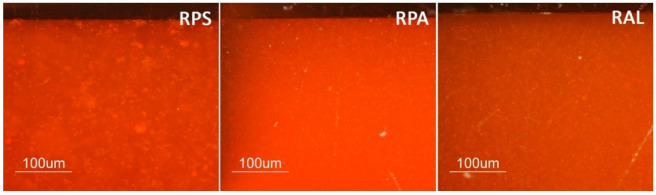
Optical microscopy (OM) images of cross-sections of the different red PMMA samples before artificial aging. Images acquired under reflected light and cross-polarizing filters at 200× original magnification.

**Figure 2:**
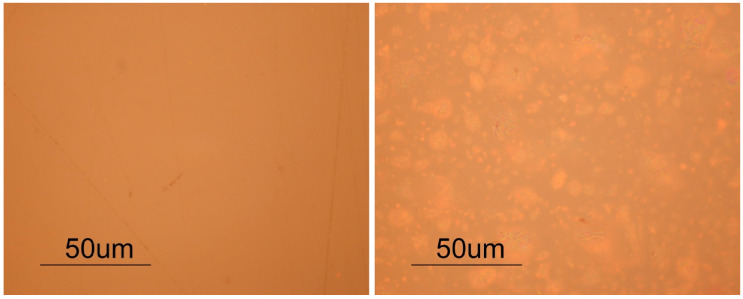
OM images of the two surfaces of RAL acquired under reflected light, bright field, at 500× original magnification. Images show that the two surfaces of the sheet are not similar, one having more accumulation of pigment agglomerates than the other. The sedimentation of the pigment might be an indication that polymerization of the PMMA sheets was conducted in horizontal position.

Observation of the transparent samples through optical microscope (OM) showed no significant features, and the surfaces from the three different sheets seemed identical. In turn, some particularities were observed in the red samples. Figure 1 presents OM images of cross-sections of the three red samples. It is visible that RPS sample presents bigger pigment agglomerates than RPA or RAL, which might indicate the use of less finely grounded pigment by Plásticos do Sado. Regarding the RAL sheet, one of the surfaces of the samples presents more accumulation of pigment than the other, Figure 2, which might indicate that polymerization of the PMMA was conducted in horizontal position allowing the pigment to sediment. Although in both industrial methods studied in Portugal the polymerization was mentioned to have been conducted in vertical orientation, the horizontal position was also common [36,37,38] and agrees with the description of the process for Altuglas^®^ production in [4,50].

Table 3 presents the average values and respective standard deviation for colorimetric measurements (L*, a*, b*) on the test samples. The values obtained for the transparent samples result from the white paper used as base during measurements, with the goal of detecting and following an eventual yellowing during the artificial aging experience. Therefore, the lower L* value of TPL when compared to the other transparent samples before aging, may be attributed to the higher thickness of these samples, which results in a greater barrier for light reflection on the white paper. In turn, the lower L* value obtained for the RPA sample reflects its visible darker color when compared with the other red samples. The higher standard deviation associated with b* coordinate obtained for RAL samples is most probably related to the differences between top and bottom surfaces (Figure 2).

#### 3.1.2. Molecular Characterization

**Figure 3:**
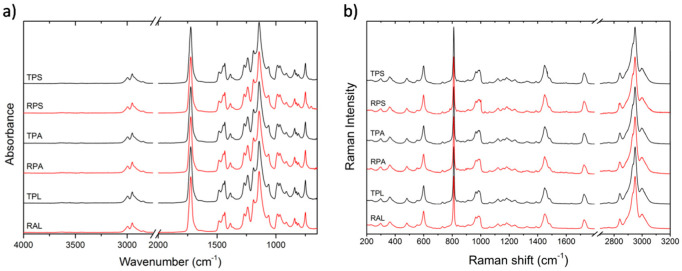
Infrared (FTIR-ATR) (**a**) and Raman (**b**) spectra of all the transparent and red samples, before artificial aging; all spectra present a similar profile, which corresponds to PMMA homopolymer.

Polymer matrixes were characterized by FTIR-ATR and Raman spectroscopies, Figure 3. PMMA homopolymer is identified in all samples by the strong C=O stretching absorption peak at 1723 cm^−1^ and the characteristic profiles and relative intensities of the other diagnostic peaks, namely the C–C–O stretching at 1269 and 1239 cm^−1^, C–O–C stretch at 1190 and 1143 cm^−1^, and C–H stretching at 2995, 2951, and 2843 cm^−1^in the FTIR-ATR spectra. In the Raman spectra, the correspondent C–H stretching vibration peaks are visible around 2996, 2949, and 2842 cm^−1^, as well as the carbonyl stretching at 1727 cm^−1^. The other dominant bands in the Raman spectra are the C–H bend at 1450 cm^−1^, the C–O–C stretching at 811 cm^−1^ and C–C–O stretching at 599 cm^−1^ [51,52]. No other peaks that could be attributed to additives have been detected. This was expected since additives in PMMA sheet production are normally used in small amounts [53,54] and therefore may be present below the detection limits of these techniques. Alterations in the typical absorptions that could be related with degradation of the polymer in the artist’s samples (TPL and RAL) were also not visible.

**Figure 4:**
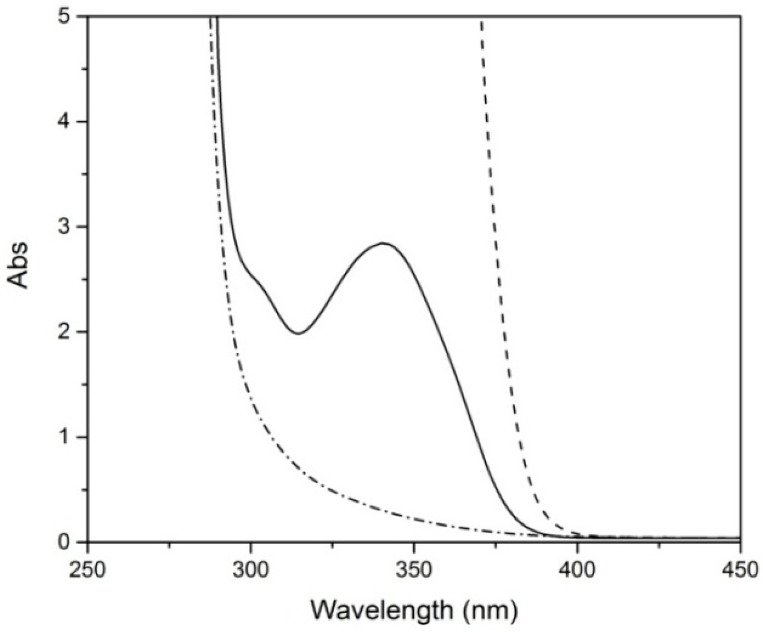
UV-Vis spectra of transparent PMMA samples; TPS (dashed line), TPA (solid line) and TPL (dash-pointed line) before artificial aging.

Figure 4 presents the UV-Vis spectra of the three types of transparent samples before artificial aging. It is known that pure PMMA absorbs mainly wavelengths <260 nm [15,16,17,18,19,20,55], but thick samples may present absorption until 300 nm due to the accumulation of chromophores, present in the polymer matrix as consequence of processing [19]. This is probably the case of TPL; however, the additional absorption at 300–400 nm observed in the UV-Vis spectra of TPS and TPA samples is most probably associated with the presence of additives. PMMA for standard use is usually modified with additives, namely UV absorbers to protect from aggressive UV radiation [56]. The different profiles reflect different sheet formulations, which were studied further by mass spectrometry.

TD-GC/MS analysis allowed the identification of several additives such as plasticizers, UV stabilizers, and release agents on the PMMA samples; a summary of the results obtained is shown in Table 4.

The initiator azobisisobutyronitrile (AIBN) was detected in all samples, which confirms the information collected regarding Plásticos do Sado and Paraglas during the historical research [11], presented in Table 1. Different plasticizers were detected in the samples; all were identified as phthalates, the most widely used plasticizers worldwide [57]. Bis(2-ethylhexyl) phthalate (DEHP), a structural (or constitutional) isomer of dioctyl phthalate (DOP), was detected in Plásticos do Sado samples (both TPS and RPS) and also in the artist’s sample TPL. Its identification in Plásticos do Sado also agrees with the information gathered in the historical research [11]. In the other artist’s sample, RAL, the plasticizer identified was dibutyl phthalate (DBP). In Paraglas samples, two different plasticizers were detected: Diisononyl phthalate (DINP) in TPA, and DBP in RPA. Additionally, traces of diethyl phthalate (DEP) were detected in all samples. Although a quantitative analysis of the additives was not performed in this study, it is possible to infer from the chromatograms the relative amount of plasticizer in the different PMMA typologies by comparing the ratio between plasticizer and MMA peaks (the main volatile). When evaluating the reference samples, it is clear that more plasticizer was used in PMMAs by Plásticos do Sado than by Paraglas. In the artist’s samples, higher amount of plasticizer was detected in TPL than in RAL but this difference is not so marked. UV-stabilizers were found only in two reference PMMAs: Etocrylene (ethyl 2-cyano-3,3-diphenylacrylate) in TPS and drometrizole (2-(2-hydroxy-5-methylphenyl)benzotriazole) in TPS and RPA. The reason for using two different UV-stabilizers in the same PMMA sheet, TPS, was not clarified. Palmitic and stearic acids were detected in trace in all samples except TPA; these compounds are normally used as lubricants or release agents, facilitating the removal of PMMA sheets from the molds. The use of stearic acid as release agent in Plásticos do Sado agrees with the data collected during historical research.

Molecular weight data obtained by SEC are presented in Table 5. In what concerns the results for the test samples before artificial aging (0 h), all weight-average molecular weight (*M*_w_) values fall in the order of millions (10^6^) confirming that all the sheets were cast [38]. Comparing the samples produced by Plásticos do Sado to the ones produced by Paraglas, it is visible that the first have somewhat lower molecular weights and higher molecular-weight dispersity (*Đ*_M_) values. This might be explained by two factors: The use of recycled monomer (that may introduce impurities that act as “chain-transfer agents” which short-stop the polymer growth locally and create a more irregular structure [58] and the lack of a post-polymerization step (residual monomer may also contribute to a more irregular structure). Interesting is also the difference between the two artist’s samples (TPL and RAL). TPL presents similar *M*_w_ and *Đ*_M_ values to the ones obtained for TPS, while RAL presents the highest molecular weight and the lower dispersity of all samples. This difference may be explained by different productions processes (unknown to the authors) as it seems to be the case with the Portuguese samples. Even so, considering that both samples have already more than 50 years of natural aging and that PMMA degrades mainly by chain scission [21], these values are an indication of the great stability of both PMMA sheets.

#### 3.1.3. Mechanical Characterization (Via Vickers Hardness)

**Figure 5:**
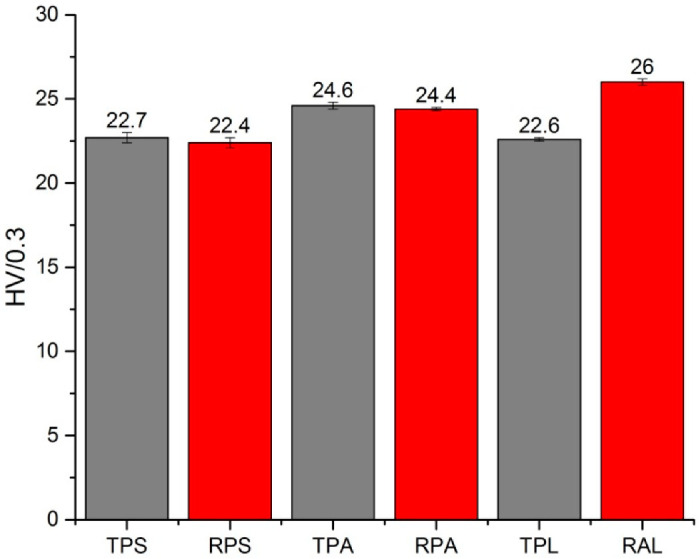
Average Vickers hardness values obtained for all PMMA samples before artificial aging and respective error bars.

Vickers Hardness (HV) values obtained for the test samples before artificial aging are reported in Figure 5. The presence of pigments and eventual fillers in the sheets does not seem to be a determinant factor for their superficial hardness, since no tendency that separates transparent from red opaque samples is observed. The same applies for the age of the sample, as the two artist’s samples, both with 50–60 years old, present the highest and one of the lowest HV values, 26 for RAL and 22.6 for TPL, respectively. Again, the production method seems to be the main factor influencing the surface hardness of PMMA sheets, as both samples (transparent and red) from each Portuguese factory present similar values, being the material by Paraglas clearly harder than the one by Plásticos do Sado. What are the aspects in the production that influence the hardness? One important aspect seems to be the plasticizer. As expected, samples with more plasticizer (Table 4) are softer (lower HV) than the others. However, Altuglas sample (RAL) apparently has more plasticizer in its composition than the sheets produced by Paraglas (TPA and RPA) but is harder. This difference is most probably due to the higher molecular weight of RAL sample (Table 5). Samples with lower molecular weights present also the lower HV values. The data obtained illustrates that the surface hardness of the PMMA sheets results from the combination of these two factors, amount of added plasticizer and molecular weight.

#### 3.1.4. Thermal Stability

**Figure 6:**
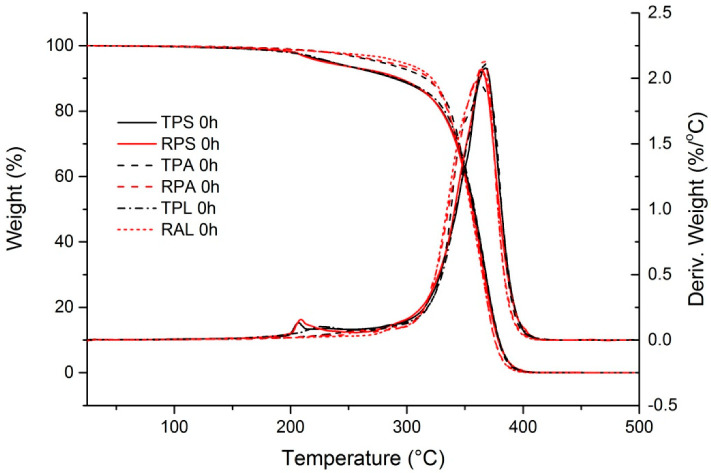
TG and DTG curves of PMMA samples before artificial aging. Curves obtained at a heating rate of 10 °C/min, under a N_2_ atmosphere.

The thermogravimetric weight loss curves (TG) and the weight loss derivative curves (DTG) of both red and transparent PMMA samples are presented in Figure 6; the respective thermal parameters are presented in Table 6. All samples show a severe decrease in weight between 320–400 °C, with a maximum decomposition rate at 362–368 °C, as seen by the sharp peak on the DTG curves. This corresponds to PMMA thermal degradation via chain end initiation or a mixture of chain end with random chain scission initiation [59], leading to complete unzipping of the polymer chain into MMA. Because MMA volatizes, the weight drops off to 0% after this step for all the samples. Samples TPA, RPA, and RAL degrade in this single step, while samples TPS, RPS, and TPL present a previous degradation step at around 200 °C.

Thermal degradation of PMMA with more than one step has been reported by several authors [43,60,61,62,63,64] and the lower temperature degradation steps are attributed to internal structural defects created during polymerization such as unsaturated-end groups (vinylidene ends produced by disproportionation termination), head-to-head links (produced by recombination termination), and/or peroxides (formed when polymerization occurs in the presence of oxygen), which all have thermally labile links. Hirata and co-workers [60] have concluded that volatilization of impurities, such as unreacted initiator or residual monomer, may be responsible for weight losses at temperatures below 210 °C by comparing the TG and DTG curves of a commercial PMMA sample before and after purification. In addition, it is known that the presence of additives may also influence the thermal behavior of the samples [64,65].

Several aspects may contribute to the differences in the thermal behavior of Plásticos do Sado samples (TPS and RPS) in what relates to the first degradation step (around 206 °C). The presence of impurities may be related with the use of recycled monomer. In turn, residual monomer (if still present) is due to the lack of the post-polymerization step. Considering the composition of the samples (Table 4), the presence of more plasticizer (also observed in TPL) may also play a role. However, in these PMMA sheets, plasticizers are present in small quantities (not detectable by FTIR-ATR) and since the plasticizer DEHP decomposes at temperatures close to PMMA, around 323 °C [65], the first degradation step cannot be directly related with the volatilization of plasticizer. However, if the presence of plasticizer in the polymer matrix reduces the inter-chain interactions [57] it may influence its thermal behavior originating a less resistant structure. The fact that the same plasticizer, DEHP, was identified in Plásticos do Sado samples and in the artist sample TPL raises the question if not only the quantity used but also the nature of the plasticizer may have an influence on thermal degradation, but with the present data it is impossible to reach a conclusion. Nevertheless, TG curves of Plásticos do Sado reveal the presence of more structural defects in the polymer chains of these samples, which are most probably associated with the use of recycled monomer and the lower control of the polymerization. On the contrary, the use of a chain transfer/termination agent [66] and the performance of a post-polymerization step by Paraglas result in fewer thermally instable links in the PMMA structure, and therefore the first stage of thermal degradation of the polymer is not present. Interestingly, the presence of Cd(S,Se) pigment does not seem to have an influence in the thermal degradation of the polymer in the reference samples.

Regarding the artist’s samples, Plexiglas sample (TPL) shows a similar behavior to Plásticos do Sado ones, while Altuglas sample (RAL) is closer to the Paraglas ones. It seems that 50 years of natural aging did not affect the thermal stability of the samples, confirming the incredible stability of PMMA. Once more, differences observed are probably related to the presence of additives and the control of the polymerization. It is also worth notice that higher molecular weight implies less end groups potentially labile in which the chain scission can initiate; this might be contributing to the superior thermal stability of RAL compared to TPL.

#### 3.1.5. Short Note Regarding the Characterization of the Artist’s Samples

The differences observed between the two artist samples, TPL and RAL, were surprising. Given the common origin of Plexiglas and Altuglas in France [48,50], it was expected that the technologies used by the two companies would be similar, and consequently, the materials produced. Several aspects should be considered at this point which may contribute to these differences: (1) The two sheets have different thicknesses which may influence the properties of the material; (2) both Rohm & Haas and Altulor produced different types of PMMA cast sheets for different applications, therefore, we may not be comparing materials of similar grade; (3) the origin of the sheets is based exclusively in the notes of the artist, and thus subject to errors.

### 3.2. Assessment of Aging Behavior

#### 3.2.1. Molecular Alterations

As expected, molecular alterations in the polymer were clearly observed by SEC analysis. The evolution of *M*_w_, *M*_n_, and *Đ*_M_ along the artificial aging for the test samples is presented in Table 5.

Regarding the reference samples, the first aspect that can be noted is that all four PMMA sheet typologies have experienced a severe decrease in their *M*_w_ (one order of magnitude), visible already after 500 h of irradiation even though radiation with λ ≥ 300 nm (just in the absorption limit of PMMA) was used for artificial aging. Furthermore, during sample preparation for SEC analysis, no gel formation was observed, which confirmed that no crosslinking took place during irradiation, in accordance with the findings of other authors [12,23,25]. These results have also confirmed that, as expected [14,18,21], chain scission was the main photodegradation mechanism for all PMMA samples. Regarding dispersity, no significant variations were observed along the aging, except in RPS that presents a strong increase in the *Đ*_M_ value.

**Figure 7:**
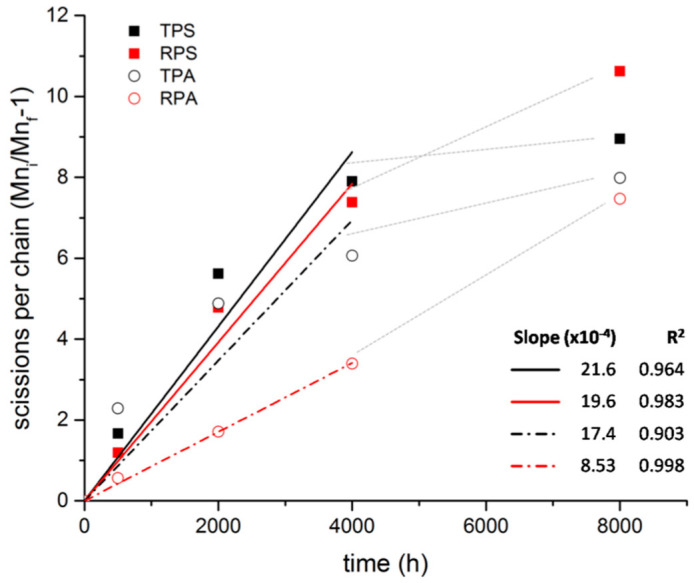
Scissions per chain of reference samples as a function of irradiation time and respective linear fit for 0–4000 h; gray lines indicate an inflection on the evolution with time.

For an easier comparison of the different PMMAs photo-stabilities, the number of scissions per chain was calculated and plotted against irradiation time. A linear fit was performed until 4000 h and the respective parameters are presented in Figure 7. The values obtained are higher than in previous studies [12,30] which may be explained by the different conditions used during the SEC analysis and in the artificial aging, namely the higher temperature inside the aging chamber.

With the exception of RPA, degradation appears to happen in two phases with two different rates, with a shift around 4000 h, being the degradation rate higher in the first phase than in the second. Siampiringue and co-authors [19] have followed the photooxidation of PMMA sheets at long wavelengths (λ > 300 nm) by FTIR and UV spectroscopies and concluded that photooxidation of PMMA is controlled by external chromophores (contaminants) which do not form new chromophores on the polymer structure during photooxidation and, therefore, the photo-reaction stops when the external photo-inductor is consumed. Although in the present study the photooxidation did not stop, as we continue to observe chain scissions, the slowdown of the degradation rate might be explained by the reduction/consumption of these external chromophores. Another explanation would be that the new oxidized groups formed by the chain scission would participate in other reactions and therefore not contribute significantly to new chain scissions.

Paraglas samples appear to be more resistant to photodegradation (less chain scission) than Plásticos do Sado samples. If the photostability of PMMA is controlled by the presence of contaminants, this difference between the two samples may be explained by the presence of more additives (Table 4) or by the use of recycled monomer (Table 1) by Plásticos do Sado.

When comparing samples from the same company, it is possible to observe that, with exception of RPS at 8000 h of irradiation, red opaque samples performed better than the transparent ones. This may be an indication of some protection/stabilization effect from the Cd (Se,S) pigment and/or BaSO_4_. Cadmium red is considered a very stable pigment [32] but to the best of our knowledge, there are no studies that have specifically looked at the influence of this pigment on the photostability of PMMA. Pintus et al. [67] have studied the influence of several inorganic pigments, including cadmium red, on the photooxidative stability of an acrylic emulsion binding medium, p(*n*BA/MMA), after UV aging. Their thermal analysis results seem to show that paint samples with cadmium red exposed to UV radiation were less prone to chain scission than the other pigmented paints tested, but they did not compare it with the binding medium alone. It is known that the photostability of a polymer system depends not only on the chemical and physical natures of the polymer and the pigment, but also on the additives present and the possible interactions between them [31] and therefore the same pigment may have different effects in different polymeric systems [68]. The change of tendency observed for the RPS sample at 4000–8000 h of irradiation must result from some not identified alteration that occurred in the polymer system during that period.

Regarding the artist’s samples, several observations can be made. First, chain scission was also the degradation mechanism observed. Second, the two samples do not present a behavior that may be considered significantly distinctive from the reference sheets. This means that the fact that these samples have already 50 years of natural aging, does not seem to be a significant factor in the way they have responded to artificial aging through photodegradation. Third, in these samples, as it was also observed for the reference ones, the decrease in molecular weight was not linear, being faster in an initial phase. In RAL the shift happens around 4000 h, but in TPL it happens before, at 2000 h, when it seems to stabilize until 4000 h, then continuing again. Even so, after 8000 h, the observed number of scissions per chain was similar for the two samples, c.10.8, and very close to the highest value observed for the reference samples, 10.6 (for RPS).

Gravimetric analysis did not show significant variations that could be clearly related with the loss of volatiles as result of photooxidative degradation. After 8000 h of artificial aging, both reference and artist’s samples had lost weight, but alterations were under 0.4% of original weight (maximum value measured for the RPS sample).

Alterations were also not detected by FTIR-ATR and Raman spectroscopies in any of the samples. The shape of the peaks and their relative intensities remained unchanged, without formation of new shoulders and/or bands both in infrared and Raman spectra of the aged samples. In the one hand, since PMMA degraded through chain scission, the degradation products maintain basically the same molecular structure except additional termination groups. With the high molecular weights of cast sheet, the proportion of the additional termination groups to the main structure would be insufficient to produce visible changes in the spectra. On the other hand, because additives were used in quantities under the detection limit of these two techniques, it was impossible to follow their eventual alteration or loss during aging.

**Figure 8:**
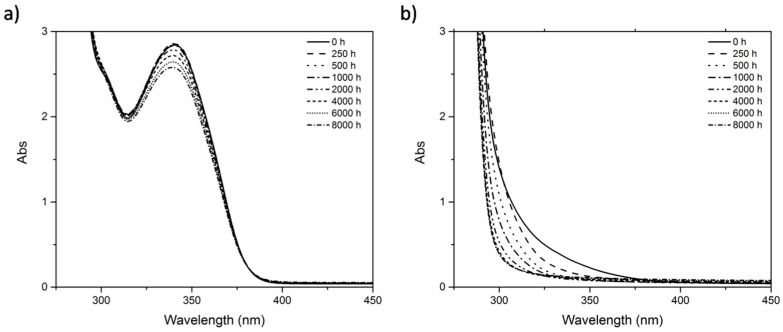
Absorption spectra of: (**a**) Reference sample TPA and (**b**) artist sample TPL, during artificial aging.

By UV-Vis spectroscopy, however, it was possible to detect some alterations. Regarding the reference samples, alterations in the polymer matrix were not detected because the absorption by additives in the 250–400 nm interval masks the region where alterations on the PMMA could be expected [13,16,19,23]. However, in TPA it was possible to follow the decrease of the band at 340 nm between 2000 and 8000 h of artificial aging (Figure 8a), which indicates that the additive responsible for this absorption is being consumed/released during irradiation exposure. In TPS, the absorption in these wavelengths is so high that saturates the signal and eventual alterations cannot be detected. Alterations in the spectra of the red samples (RPA and RPS) are related with color alteration and are presented in Supplementary Materials.

Regarding the artist’s samples, a decrease in absorption in the tail at 280–400 nm in TPL is observed (Figure 8b). This is interesting because it is the opposite of what was observed by other authors [19], which have correlated an increase in this region with the formation of photoproducts during the photooxidation of the extrinsic chromophores. Would the decrease observed in our case be related with the consumption/volatilization of those photoproducts, which were maybe formed during the natural aging? Another possible explanation would be the loss of additives, which also absorb in this region. Interestingly, this alteration only happens in the first 2000 h, after that, between 4000 and 8000 h, the absorption is stabilized, which means that whatever was being consumed has disappeared.

#### 3.2.2. Visual Alterations

Alterations on the test samples were also followed by colorimetry and results are presented in Table 3. For the reference transparent samples, ∆E* values after 8000 h of artificial aging are around 1.5 for both TPS and TPA, which is a variation under the JND (just noticeable difference) limit of 2.3 [69]. However, for artist sample TPL, a ∆E* value of 2.6 was calculated, which is above the JND and reflects mainly a decrease in the L* coordinate. This higher ∆L* value is probably due to its greater thickness and its aging. Variations in red samples are more significant, especially in RPS samples with a ∆E* = 11.59. This value reflects mainly a variation on the b* axis towards a less saturated color. Even so, it is possible to confirm the excellent lightfastness of the cadmium red pigment after such a long period of irradiation. The red artist sample, RAL, presents a variation higher than what was observed for the reference sample RPA but half of the alteration on RPS. Apparently, the 50 years of natural aging of the artist’s sample did not have a negative effect on its aging performance, in what concerns the visual parameters observed. More data regarding color alteration in red samples is reported in Supplementary Materials.

In addition, alterations on the samples were followed by OM by photographing the same areas of the samples along the aging experience.

As observed by the other techniques, results show differences in the photostability of the reference samples from Plásticos do Sado and Paraglas.

**Figure 9:**
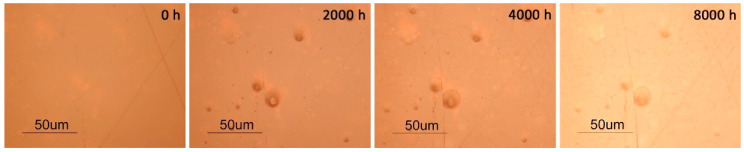
OM images of the surface of an RPS sample during artificial aging; acquired under reflected light and in bright-field mode.

In RPS, the formation of several bubble like features in the interior of the sample became evident after 2000 h of irradiation. These features, probably formed by volatile photodegradation products, have enlarged and/or have migrated to the surface afterwards, as visible in Figure 9. The same phenomena seemed to have started in the TPS samples after 6000 h, but in a much minor degree. This was not observed in the Paraglas samples.

**Figure 10:**
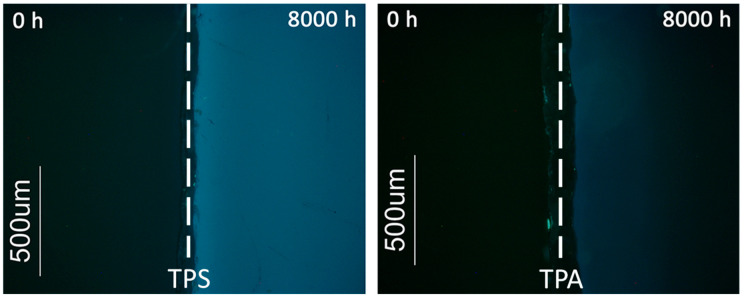
Fluorescence microscopy images under blue-violet light of TPS and TPA surfaces. A non-aged sample (0 h) and an artificial aged sample (8000 h) of each PMMA typology were photographed side-by-side. Both PMMA sheets presented no fluorescence before artificial aging (dark surfaces at 0 h); TPS developed more fluorescence (lighter blue under radiation) than TPA during aging.

Another important aspect was the development of auto-fluorescence in the transparent samples during artificial aging. Auto-fluorescence was observed when samples were illuminated with blue-violet light (λ = 395–440 nm), Figure 10. Studies that use fluorescence microscopy as a tool to follow polymer degradation are scarce [70] and none was found that specifically follows PMMA photodegradation. The development of fluorescence in artificially aged PMMA samples was detected by fluorescence spectroscopy by Dickens and co-workers [28], with a maximum excitation at ~375 nm and emission at 480 nm, attributed by the authors to the formation of carbonyl groups in α-diketones and α,β unsaturated aldehydes. Other authors [19] have observed an increase in the absorption spectra between 280–500 nm in commercial acrylic sheets artificially aged; the absorption at λ > 400 nm was related with the photoproducts that resulted from photooxidation of extrinsic chromophore contaminants in the polymer matrix. Probably similar photoproducts are responsible for the observed fluorescence in the samples within the present study. This phenomenon is more pronounced in the TPS samples, which indicates that this sheet is more prone to photooxidation, even if only by the extrinsic chromophores. This agrees with the results obtained with the other techniques.

Observation by OM of the artist’s PMMA samples could only detect alterations on TPL. After 6000 h of irradiation, small features seem to have started to form in the interior of the sample with a similar appearance and degree to what was observed on TPS after the same period of irradiation. Additionally, a micro-crack was observed on the top TPL surface and several on the bottom. There was not a significant development of auto-fluorescence. In RAL no alterations were observed after 8000 h of artificial aging.

#### 3.2.3. Mechanical Alterations (Via Vickers Hardness)

**Figure 11:**
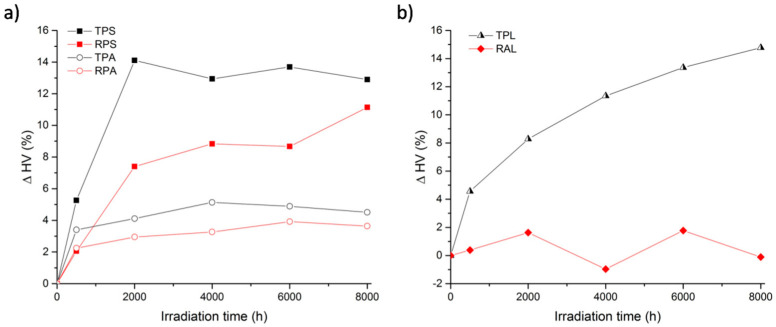
Vickers hardness values variation versus irradiation time for: (**a**) Reference samples (TPS, RPS, TPA, and RPA); and (**b**) artist’s samples (TPL and RAL).

Figure 11a shows the variation of HV in percentage versus irradiation time, for both transparent and red reference samples. The first aspect worth notice is the increase in surface hardness with artificial aging in all four samples. This is curious since SEC analysis has shown a decrease in molecular weight as result of chain scission, expected to correspond to a decrease of the mechanical strength. A similar increase in surface hardness (in nano-scale) was observed by other authors [71] for PMMA exposed to UV radiation, who have related this behavior with a relaxation process leading to a more compact reorganization of the macromolecules of the top layer. Another factor that may be responsible for the increase in hardness in the samples under study, could be the loss (or further reactions) of plasticizers at the surface level. This would explain why Plásticos do Sado samples, which have more plasticizer (see Table 4), present a stronger increase in surface hardness than the ones by Paraglas. Another interesting aspect is that apparently, in both brands, red samples seem to have suffered less superficial alterations than the transparent ones. This tendency was also partially observed for the molecular weight alteration, reinforcing that the presence of the red cadmium pigment and/or the filler might be having a protection role in the system against photooxidation.

In Figure 11b is possible to follow the variation of the surface hardness of the artist’s samples along the artificial aging. As can be observed, the two samples show very different behaviors. While in RAL the surface hardness is kept almost stable during artificial aging (variation is less than 2%), in TPL the surface hardness is increasing continuously. It seems that after 8000 h of aging, this already naturally aged sample is still in a process of surface reorganization. Several tentative explanations of this phenomenon could be discussed; one could be related with the loss of plasticizers, which in this thicker sample may take longer. Both behaviors are different from what was observed for the reference samples, in which after an initial period of increase in surface hardness, the samples seemed to stabilize.

#### 3.2.4. Alterations on Thermal Stability

**Figure 12:**
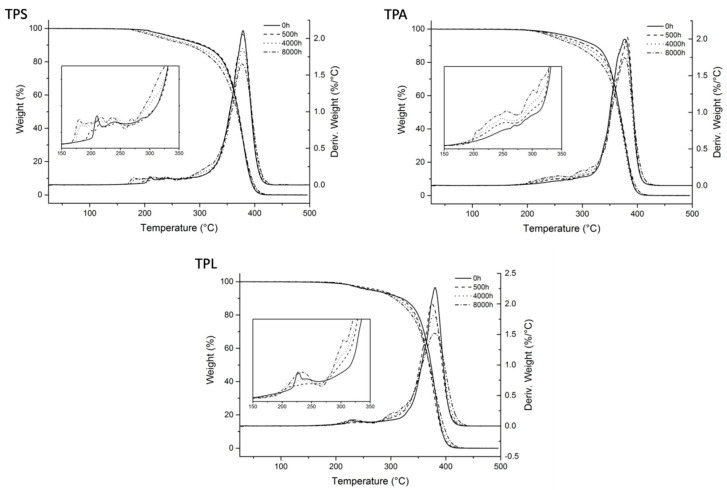
TG and DTG curves of the transparent samples after 0, 500, 2000, and 8000 h of irradiation.

In Figure 12 are presented the TG and DTG curves of the transparent samples after different irradiation times. A common feature in both reference samples (TPS and TPA) is that, with aging, degradation starts at lower temperatures, even though the temperature of maximum decomposition rate remains the same. This shift for lower temperatures reflects the presence of more thermally labile links, which are formed during the photooxidation reactions, in particular as result of chain scission.

As observed for the reference samples, also the artist’s sample TPL shows an increase of volatiles at lower temperatures with artificial aging. But even though the thermogram of the sample before aging was similar to the reference sample TPS, in this case, a shift to temperatures lower than 200 °C was not observed.

## 4. Conclusions

In this work we have studied the impact of specific intrinsic factors, as additives and industrial production processes, on the properties and susceptibility of PMMA cast sheets to photooxidtion.

The data collected showed that equivalent PMMA cast sheets produced by different companies in the same period (2000s) presented different properties such as molecular weight distribution, surface hardness, or thermal behavior. Differences were also observed in the behavior of the samples during artificial aging. Namely, Plásticos do Sado samples showed a higher degree of scissions per chain, higher increase in surface hardness, thermal degradation at lower temperatures, appearance of bubble like features inside the sample, and a higher development of UV fluorescence, when compared to Paraglas samples. These differences were correlated with the presence of more plasticizer in the sample composition (Table 4) and more defects in the polymeric structure due to use of recycled monomer and the lack of a post polymerization step (Table 1). The presence of the inorganic pigment Cd(S,Se) did not seem to influence the initial properties of the PMMA sheets but might have a protective effect during photodegradation, as seen by SEC analysis and micro-hardness measurements. To the best of the authors’ knowledge, it was the first time that the influence of cadmium red pigment in PMMA photodegradation was accessed.

Regarding PMMA artist’s samples produced in the 1960s, it was expected that the technologies used by the two companies would be similar, and consequently, also the quality of the materials produced. However, the behavior of the Plexiglas sheet was closer to the Plásticos do Sado reference sheet than to the Altuglas one. This is most probably related with their similar compositions, particularly in what refers to plasticizers, as shown by TD-GC/MS analysis. Nevertheless, unknown differences between the production processes of the two artist’s samples cannot be ruled out either.

Surprisingly, we did not detect major differences between references from 2000s and historical artist’s samples from 1960s. Therefore, with these results, we can conclude that the global production process (which may include the polymerization conditions, the monomer origin and/or the organic additives added) plays a more important role in the properties and aging behavior of the PMMA cast sheets than the presence of cadmium red, or the production decade of the acrylic sheets. This confirms our original hypothesis and the concerns of Lourdes Castro in the 1960s, by consciously choosing to buy what she thought were the best brands of PMMA sheet to use in her artworks.

Relevant characterization methods for assessing intrinsic properties of PMMA to help estimating their photostability were size exclusion chromatography, micro-indentation, thermogravimetry, and thermodesorption-gas chromatography/mass spectrometry.

Further research is still necessary for deeper understanding mechanisms behind the differences observed, but the results obtained show the importance of considering that not all the PMMA cast sheets are alike. This is a significant aspect when selecting testing samples for research in conservation of PMMA, and especially when considering conservation strategies for artworks made of this material. In Museum collections, the origin of the materials is most of the times unknown, but our work has proved that historical and material research may give important clues to evaluate their stability.

## Figures and Tables

**Table 1:** Main characteristics of the production of poly(methyl methacrylate) (PMMA) sheets in the two different plants (adapted from [11]).

	Plásticos do Sado	Paraglas
Monomer	Produced by the company through chemical recycling (depolymerization by pyrolysis) of acrylic scrap ^1^	Pure monomer acquired from Degussa (Germany) or Repsol (Spain)
Solution poured into the molds	Pre-polymerized syrup (degree of polymerization empirically tested, prepared by heating monomer with initiator AIBN) + colorants and additives	Monomer + initiator (AIBN) + colorants and additives
Polymerization	Polymerization in water tanks. Molds placed vertically in water tanks and heated to 50–60 °C	Rostero process [39,40,41]. Polymerization in a chamber with pressure and temperature control. Molds held vertically between metallic plates and heated to 75–80 °C
	Polymerization completed in water tanks at higher temperature (but <100 °C)	Polymerization completed in the chamber at 120 °C (post-polymerization)

^1^ From cutting remains or end of life material. Several of the recycling steps were controlled visually, based on the empirical knowledge of the workers.

**Table 2:** Identification codes of the test samples and corresponding details.

Code	Image ^a^	Producer	Date of Production	Description	Colorants ^c^	Thickness (mm)	Notes
TPS	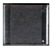	Plásticos do Sado (PT)	2000s	Colorless Transparent	n.a.	3.12 ± 0.12	From a sheet fragment with protection film.
RPS	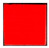			Red Opaque	Cd (S,Se)	2.88 ± 0.01	From a color swatch. One small piece.
TPA	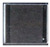	Paraglas (PT)		Colorless Transparent	n.a.	3.57 ± 0.10	From a sheet fragment with protection film.
RPA	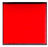			Red Opaque	Cd (S,Se)	2.87 ± 0.01	From a color swatch. One small piece.
TPL	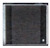	Plexiglas (DE?) ^b^	1960s ^b^	Colorless Transparent	n.a.	4.20 ± 0.12	From a sheet fragment with protection paper.
RAL	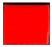	Altuglas (FR) ^b^		Red Opaque	Cd (S,Se)	2.80 ± 0.02	

^a^ Photographs taken on top of a black paper; ^b^ Samples provided by Lourdes Castro; date and brand were indicated by the artist; ^c^ Identification by XRF, SEM-EDS and Raman [11].

**Table 3:** Average values and standard deviation for color coordinates (L*, a*, b*) of the test samples, before and after 8000 h artificial aging, and respective variation.

	0 h			8000 h			Variation			
	L*	a*	b*	L*	a*	b*	∆L*	∆a*	∆b*	∆E*
TPS	91.16 (±0.12)	−0.25 (±0.02)	4.78 (±0.03)	89.81 (±0.17)	−0.21 (±0.02)	5.37 (±0.03)	−1.35 (±0.05)	0.04 (±0.03)	0.60 (±0.05)	1.48 (±0.03)
RPS	38.73 (±0.01)	61.33 (±0.02)	52.81 (±0.05)	40.15 (±0.01)	56.92 (±0.02)	42.19 (±0.07)	1.43 (±0.01)	−4.41 (±0.04)	−10.62 (±0.11)	11.59 (±0.11)
TPA	91.32 (±0.00)	−0.04 (±0.01)	4.14 (±0.01)	90.07 (±0.04)	−0.07 (±0.01)	4.98 (±0.01)	−1.25 (±0.05)	−0.03 (±0.02)	0.83 (±0.01)	1.51 (±0.04)
RPA	32.78 (±0.01)	56.72 (±0.01)	41.64 (±0.18)	31.93 (±0.01)	54.04 (±0.03)	40.18 (±0.17)	−0.85 (±0.02)	−2.68 (±0.04)	−1.46 (±0.12)	3.17 (±0.05)
TPL	89.75 (±0.20)	−0.30 (±0.01)	4.73 (±0.02)	87.26 (±0.21)	−0.04 (±0.02)	5.39 (±0.01)	−2.50 (±0.27)	0.26 (±0.02)	0.66 (±0.02)	2.60 (±0.25)
RAL	36.33 (±0.18)	57.74 (±0.25)	51.26 (±1.09)	39.34 (±0.04)	56.17 (±0.13)	47.28 (±0.31)	3.01 (±0.22)	−1.57 (±0.12)	−3.98 (±1.07)	5.28 (±0.77)

**Table 4:** Summary of additives identified by TD-GC/MS in the PMMA samples. For the abundance calculation: Peak area MMA/peak area_1_ < 5: xxx; 5–15: xx; 15–100: x; > 100: (x) (peak area_1_ = peak area of the compound of interest).

Compounds	TPS	RPS	TPA	RPA	TPL	RAL
Initiators						
Azobisisobutyronitrile (AIBN)	x	x	x	x	x	x
Plasticizers						
Diethyl phthalate (DEP)	(x)	(x)	((x))	((x))	(x)	(x)
Dibutyl phthalate (DBP)	/	(x)	/	x	/	xx
Bis(2-ethylhexyl) phthalate (DEHP) = dioctyl phthalate (DOP)	xxx	xxx	(x)	/	xxx	/
Diisononyl phthalate (DINP)	/	/	x	/	/	/
UV stabilizers						
Ethyl 2-cyano-3,3-diphenylacrylate (Etocrylene)	x	/	/	/	/	/
2-(2-hydroxy-5-methylphenyl)benzotriazole (Drometrizole)	x	/	/	x	/	/
Release agents						
Palmitic acid, methyl ester	(x)	(x)	/	(x)	(x)	/
Stearic acid, methyl ester	(x)	(x)	/	(x)	(x)	((x))

xxx: High abundance; xx: Medium abundance; x: Positive presence; (x): Trace; ((x)): Ultra-trace; /: Not detected.

**Table 5:** Evolution of weight-average molecular weight (*M*_w_), number-average molecular weight (*M*_n_), and molecular-weight dispersity (*Đ*_M_) of the test samples during artificial aging.

	0 h			500 h			2000 h			4000 h			8000 h		
	*M*_w_ (×10^5^)	*M*_n_ (×10^5^)	*Đ*_M_	*M*_w_ (×10^5^)	*M*_n_ (×10^5^)	*Đ*_M_	*M*_w_ (×10^5^)	*M*_n_ (×10^5^)	*Đ*_M_	*M*_w_ (×10^5^)	*M*_n_ (×10^5^)	*Đ*_M_	*M*_w_ (×10^5^)	*M*_n_ (×10^5^)	*Đ*_M_
TPS	12.25	4.32	2.8	4.08	1.62	2.6	1.66	0.65	2.6	1.48	0.49	3.1	1.15	0.43	2.6
RPS	12.31	3.46	3.6	6.50	1.58	3.9	2.94	0.59	4.4	2.17	0.41	5.3	1.78	0.30	6.0
TPA	13.30	6.20	2.1	5.12	1.89	2.7	2.31	1.05	2.2	1.98	0.88	2.3	1.73	0.69	2.5
RPA	12.58	4.99	2.5	9.04	3.19	2.8	4.70	1.84	2.6	3.00	1.13	2.6	1.55	0.59	2.6
TPL	12.12	4.61	2.6	3.19	1.39	2.3	1.25	0.57	2.2	1.39	0.56	2.5	0.95	0.39	2.4
RAL	19.08	9.39	2.0	12.13	5.27	2.3	5.19	2.05	2.5	2.29	0.83	2.8	1.99	0.80	2.5

**Table 6:** Thermal parameters obtained from the TG and DTG curves of the different PMMA samples, including onset temperature (*T*_0_) and maximum process rate temperature (*T*_max_) in °C, and respective mass losses (∆_m_) in % of original mass, for the two thermo degradation steps observed.

	Initial Step		Main Step	
	*T*_0_/∆_m_	*T*_max_/∆_m_	*T*_0_/∆_m_	*T*_max_/∆_m_
TPS	206/2	207/2	325/18	368/70
RPS	206/2	208/3	324/17	364/66
TPA	-	-	328/14	362/60
RPA	-	-	325/12	367/69
TPL	215/3	224/4	333/20	367/68
RAL	-	-	324/11	367/73

## Supplementary Materials

The following are available online at https://www.mdpi.com/2073-4360/12/10/2198/s1, Figure S1: UV-Vis transmission spectrum of the filter used in the solarbox during the artificial aging experience; Figure S2: Absorption spectra of RPS and RPA during artificial aging; Figure S3: Evolution of the *a** and *b** coordinates of the red reference samples along the artificial aging.
