# Correction: Babo et al. Characterization and Long-Term Stability of Historical PMMA: Impact of Additives and Acrylic Sheet Industrial Production Processes. *Polymers* 2020, *12*, 2198

**DOI:** 10.3390/polym13234105

**Published:** 2021-11-25

**Authors:** Sara Babo, Joana Lia Ferreira, Ana Maria Ramos, Anna Micheluz, Marisa Pamplona, Maria Helena Casimiro, Luís M. Ferreira, Maria João Melo

**Affiliations:** 1 Department of Conservation and Restoration and Research Unit LAQV-REQUIMTE, NOVA School of Sciences and Technology (FCT NOVA), 2829-516 Caparica, Portugal; mjm@fct.unl.pt; 2 Department of Chemistry and Research Unit LAQV-REQUIMTE, NOVA School of Sciences and Technology (FCT NOVA), 2829-516 Caparica, Portugal; ana.ramos@fct.unl.pt; 3 Conservation Science Department, Deutsches Museum, Museumsinsel 1, 80538 Munich, Germany; a.micheluz@deutsches-museum.de (A.M.); m.pamplona@deutsches-museum.de (M.P.); 4 Center for Nuclear Sciences and Technologies (C2TN), Instituto Superior Técnico (IST), Universidade de Lisboa, 2695-066 Bobadela LRS, Portugal; casimiro@ctn.tecnico.ulisboa.pt (M.H.C.); ferreira@ctn.tecnico.ulisboa.pt (L.M.F.); 5 Department of Nuclear Sciences and Engineering (DECN), Instituto Superior Técnico (IST), Universidade de Lisboa, 2695-066 Bobadela LRS, Portugal

The authors wish to make a change to the published paper [1]. In the original manuscript, Figure 12, the first two thermograms were switched by mistake. The corrected Figure 12 is presented below.

The authors apologize for any inconvenience caused and state that the scientific conclusions are unaffected. The original publication has also been updated. 

## Figures and Tables

**Figure 12:**
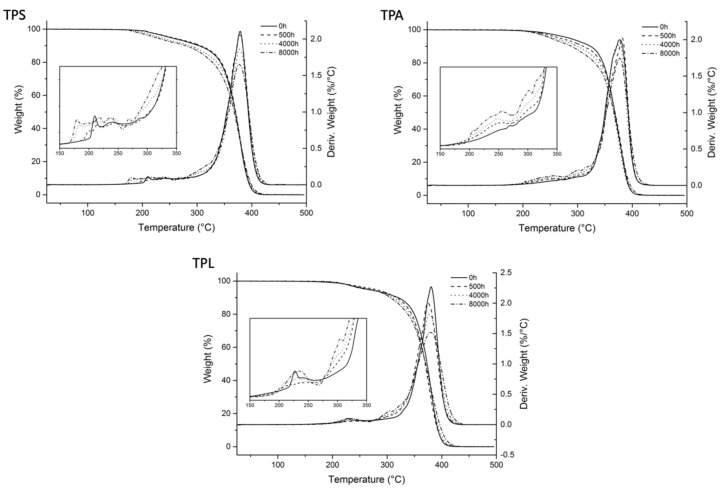
TG and DTG curves of the transparent samples after 0, 500, 2000, and 8000 h of irradiation.
