# Supplementary material for: Characterization and Long-Term Stability of Historical PMMA: Impact of Additives and Acrylic Sheet Industrial Production Processes
Source: Polymers (Basel). 2020 Sep 25;12(10):2198. doi: 10.3390/polym12102198 (PMC7601423; doi:10.3390/polym12102198)
Supplement: Supplementary file 1 [file polymers-12-02198-s001.pdf]

polymers

MDPI

Supplementary Information

Characterization and Long-Term Stability of Historical PMMA: Impact of Additives and Acrylic Sheet Industrial Production Processes

Sara Babo [1,*], Joana Lia Ferreira [1,*], Ana Maria Ramos [2], Anna Micheluz [3], Marisa Pamplona [3], Maria Helena Casimiro [4], Luís M. Ferreira [4,5] and Maria João Melo [1]

[1] Department of Conservation and Restoration and Research Unit LAQV-REQUIMTE, NOVA School of Sciences and Technology (FCT NOVA), 2829-516 Caparica, Portugal
[2] Department of Chemistry and Research Unit LAQV-REQUIMTE, NOVA School of Sciences and Technology (FCT NOVA), 2829-516 Caparica, Portugal; ana.ramos@fct.unl.pt
[3] Conservation Science Department, Deutsches Museum, Museumsinsel 1, 80538 Munich, Germany; ana.ramos@fct.unl.pt (A.M.); m.pamplona@deutsches-museum.de (M.P.)
[4] Center for Nuclear Sciences and Technologies (C2TN), Instituto Superior Técnico (IST), Universidade de Lisboa, 2695-066 Bobadela LRS, Portugal; casimiro@ctn.tecnico.ulisboa.pt (M.H.C.); ferreira@ctn.tecnico.ulisboa.pt (L.M.F.)
[5] Department of Nuclear Sciences and Engineering (DECN), Instituto Superior Técnico (IST), Universidade de Lisboa, 2695-066 Bobadela LRS, Portugal
* Correspondence: sara.sbabo@gmail.com (S.B.); jlaf@fct.unl.pt (J.L.F.)

The supporting information has the following content:

1. Transmission spectra of the filter used in the solarbox
2. UV-Vis and colourimetry results from alterations in the red PMMA reference samples

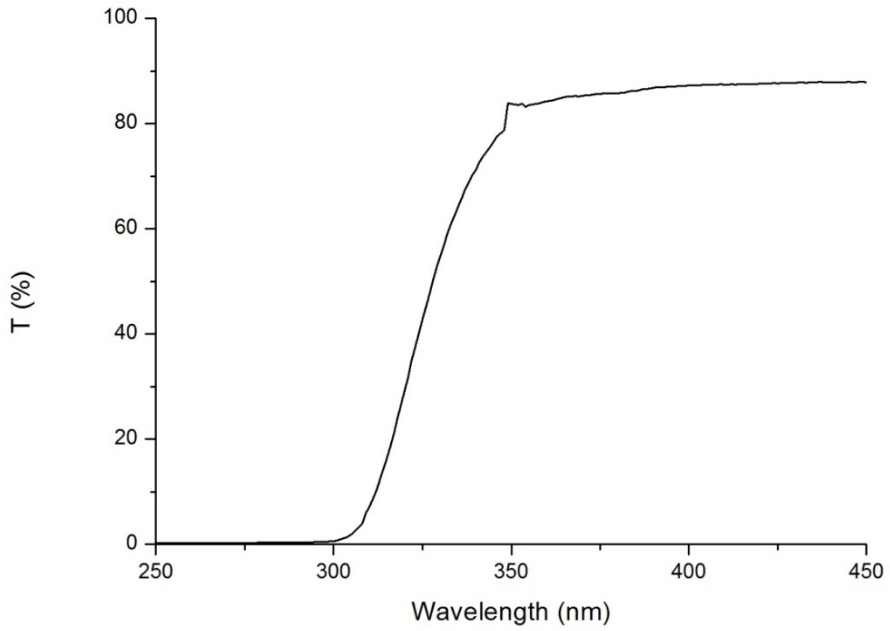

Figure S1: UV-Vis transmission spectrum of the filter used in the solarbox during the artificial ageing experience.

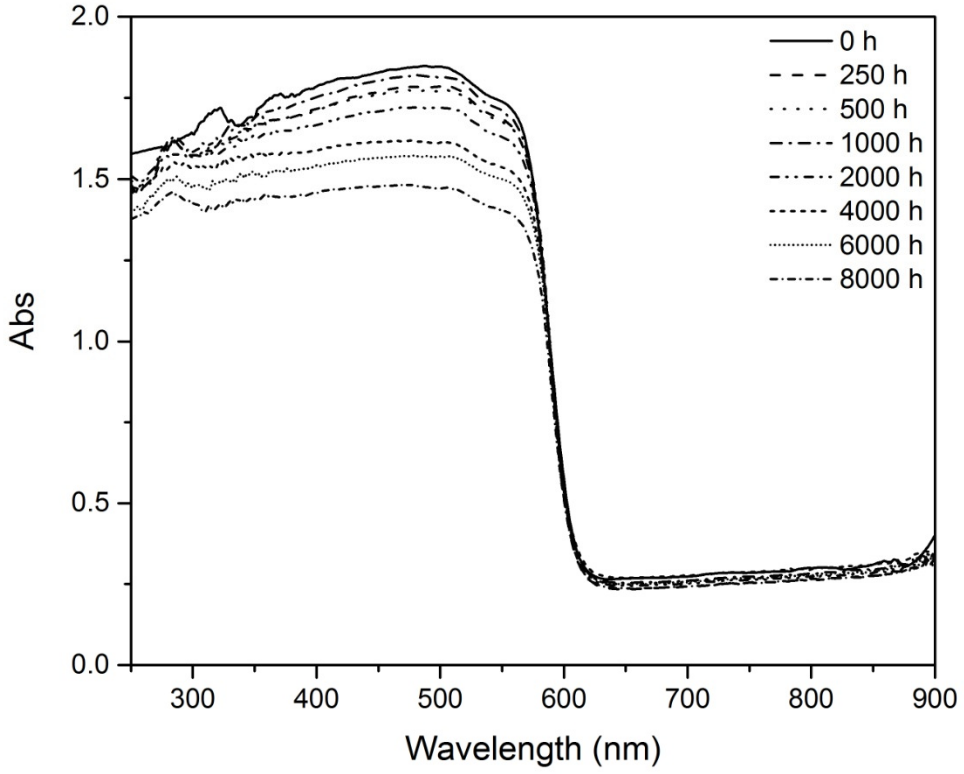

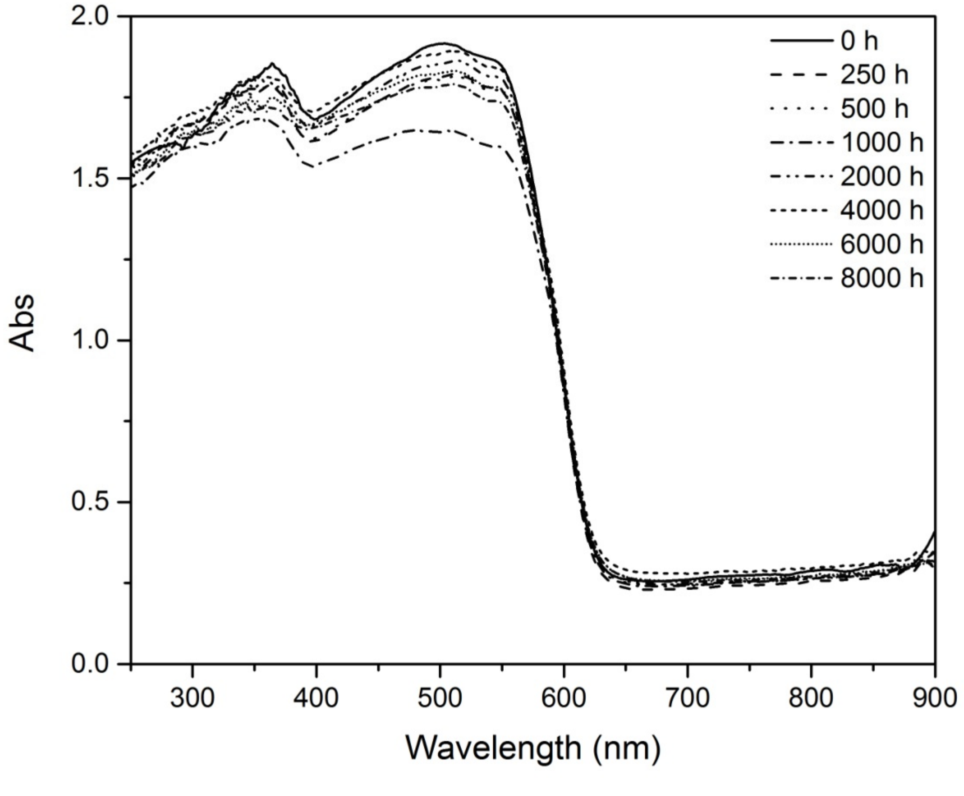

Figure S2: Absorption spectra of RPS (top) and RPA (bottom) during artificial ageing.

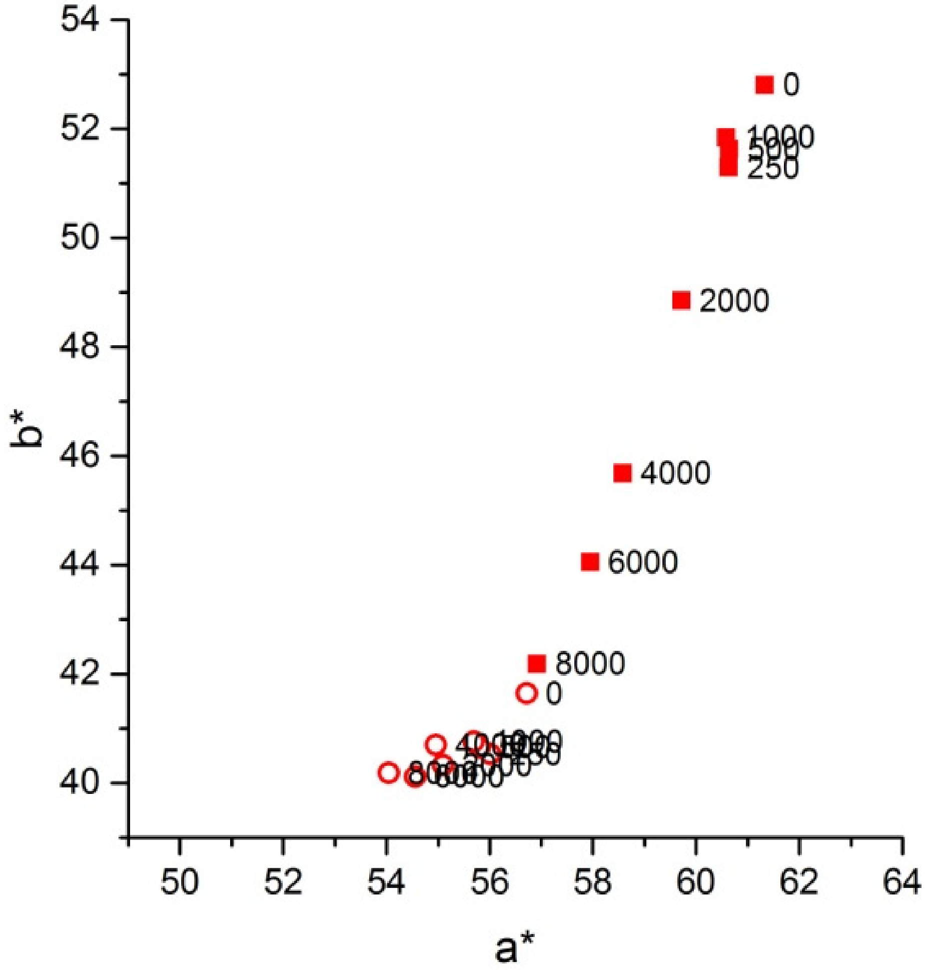

Figure S3: Evolution of the a* and b* coordinates of the red reference PMMA samples, RPS (red squares) and RPA (red circles) along the artificial ageing.

© 2020 by the authors. Licensee MDPI, Basel, Switzerland. This article is an open access article distributed under the terms and conditions of the Creative Commons Attribution (CC BY) license (http://creativecommons.org/licenses/by/4.0/).
